# Adaptive Group-combined P-values Test for Two-sample Location Problem with Applications to Microarray Data

**DOI:** 10.1038/s41598-018-26409-1

**Published:** 2018-05-25

**Authors:** Shenghu Zhang, Jiayan Zhu, Zhengbang Li

**Affiliations:** 1 School of Mathematics and Information Science, Jiangxi Normal University, Nanchang, 330022 China; 2 School of Mathematical Sciences, University of Chinese Academy of Sciences, Beijing, 100049 China; 3 School of information engineering, Hubei University of Chinese Medicine, Wuhan, 430065 China; 4 School of Mathematics and Statistics & Hubei Key Laboratory of Mathematical Sciences, Central China Normal University, Wuhan, 430079 China

## Abstract

The purpose of this article is to propose a test for two-sample location problem in high-dimensional data. In general highdimensional case, the data dimension can be much larger than the sample size and the underlying distribution may be far from normal. Existing tests requiring explicit relationship between the data dimension and sample size or designed for multivariate normal distributions may lose power significantly and even yield type I error rates strayed from nominal levels. To overcome this issue, we propose an adaptive group p-values combination test which is robust against both high dimensionality and normality. Simulation studies show that the proposed test controls type I error rates correctly and outperforms some existing tests in most situations. An Ageing Human Brain Microarray data are used to further exemplify the method.

## Introduction

In recent decades, technological advances have made it possible to collect simultaneously massive amounts of high-throughput data. For example, in biomedical studies, lots of magnetic response images (MRI) and functional MRI data are gleaned for each subject^1^; various microarray expression patterns of thousands of genes are measured^2^. In addition, examples of these kinds are plentiful in computer science, engineering, climatology, geology, and finance. This type of data, often called high-dimensional data, are characterized with a large number of variables *m* and a relatively small number of samples *n*, usually *m* is considerably large than *n* ($m\gg n$). So developing approaches for high-dimensional data is of great practical importance. In this context, a problem of concern is to test for the equality of location parameters of two samples simultaneously. Assume that $\{{X}_{i1},{X}_{i2},\cdots ,{X}_{i{n}_{i}}\}(i=\mathrm{1,}\,\mathrm{2)}$ are two independent random samples of sizes *n*_1_ and *n*_2_, from *m*-variate distributions *F*_1_(*X* − *μ*_1_) and ${F}_{2}(X-{\mu }_{2})$ with *m*-variate location parameters *μ*_1_ and *μ*_2_, respectively. We consider the following high-dimensional null hypothesis ${H}_{0}:{\mu }_{1}={\mu }_{2}$.

A traditional approach for this hypothesis is the Hotelling’s *T*^2^ test given by ${T}^{2}=\frac{{n}_{1}{n}_{2}}{{n}_{1}+{n}_{2}}{({\bar{X}}_{1}-{\bar{X}}_{2})}^{\tau }{S}_{n}^{-1}({\bar{X}}_{1}-{\bar{X}}_{2})$, where ${\bar{X}}_{1}={\sum }_{j=1}^{{n}_{1}}{X}_{1j}/{n}_{1}$ and ${\bar{X}}_{2}={\sum }_{j=1}^{{n}_{2}}{X}_{2j}/{n}_{2}$ are the sample means, *S*_*n*_ is the sample covariance matrix, and *n*_1_, *n*_2_ are the sizes of two samples. The Hotelling’s *T*^2^ test requires that the data dimension *m* is fixed and less than ${n}_{1}+{n}_{2}-2$. It possesses desirable properties for low-dimensional data when *m* is fixed. However, the situation is changed for high-dimensional data. Bai and Saranadasa^3^ studied the performance of the Hotelling’s *T*^2^ test for high-dimensional data and found that its powers drop significantly as *m*/*n* increases. A reason for this phenomenon is that Hotelling’s *T*^2^ test contains the inverse of sample covariance matrix which may not converge to the population covariance matrix when *m* is close to *n* or even is undefined when *m* > *n*.

To address this issue, under the assumption of equal covariance matrix, Bai and Saranadasa^3^ proposed a new test by removing ${S}_{n}^{-1}$ from the Hotelling’s *T*^2^ test. They also derived the asymptotic normality of the test statistic when *m* and *n* are of the same order. However, this requirement is too restrictive for high-dimensional data, in which *m* is often far larger than *n*. Motivated by this, Chen and Qin^4^ proposed to remove the squared term ${\sum }_{j=1}^{{n}_{i}}{X}_{ij}^{\tau }{X}_{ij}(i=\mathrm{1,}\,\mathrm{2)}$ from $||{\bar{X}}_{1}-{\bar{X}}_{2}{||}^{2}$ (Bai and Saranadasa’s test) which poses demands on the dimensionality but makes no contribution in testing, where $||\cdot ||$ is the squared Euclidean distance. Note that these methods are scalar-invariant since the magnitudes of variables’ variances which may vary greatly are not taken into account. Neglecting such heterogeneity information could lose power dramatically since the variables with larger variabilities which dominates the results may not be statistically significant. Hence under the assumption of multivariate normality, Srivastava *et al*.^5^ developed a scalar-transformation-invariant test by replacing *S*_*n*_ in the Hotelling’s *T*^2^ test with its diagnoal matrix. However, the aforementioned tests are essentially parametric in spirit since their performance would be degraded dramatically when the assumption of normality is not met, especially for heavy-tailed distributions. To overcome this limitation, Feng *et al*.^6^ proposed a scalar-invariant test based on multivariate-sign-based procedures which is robust against non-normality.

Although many existing methods are available to test for the equality of location parameters of two samples, most of them perform well under certain conditions on the degree of *m*/*n*. For example, Bai and Saranadasa^3^ reqiures $p/n\to c\in \mathrm{(0,}\,\mathrm{1)}$; Chen and Qin^4^ requires ${\rm{tr}}({{\rm{\Sigma }}}^{4})=o({{\rm{tr}}}^{2}({{\rm{\Sigma }}}^{2}))$, where Σ is the covariance matrix; Srivastava *et al*.^5^ needs $n=O({m}^{\delta })$ for 1/2 < δ < 1. Nevertheless, in a range of high-dimensional applications, it is hard to determine the degree of *m*/*n*. Sometimes, the data dimension *m* can be unimaginable large relative to the sample size *n*. For example, in the microarray data, tens of thousands of genes are observed on tens of hundreds of samples^2,7^.

For two-sample location problem, an alternative solution is to use univariate test which constructs marginal test for each variable first and then employs some kind of p-values combination method to accelerative accumulate the marginal signals. Common p-values combination methods include Fisher’s combined method^8^, truncated product method^9^, truncated tail strength method^10^, and adaptive rank truncated product methods^11^. Hu *et al*.^12^ pointed that the performance of these combination procedures depend heavily on the magnitudes of p-values to be combined. When the magnitudes of p-values varies, they may suffer from a substantial loss of power. To tackle this issue, they proposed a group combined p-values method (denoted by GCP) for large-scale genetic association studies. In GCP, p-values are divided into three groups first and constructed into a test statistic within each group. The final test is obtained by combing these intermediated test statistics. To use GCP, one needs to define two thresholds for p-values beforehand. However, when the number of marginal tests is large, the performance of GCP is very sensible to the selection of thresholds. Hu *et al*.^12^ used two self-defined thresholds which may result in power loss when most of the investigated p-values are not included in their pre-defined groups.

In this article, we aim to propose an adaptive group p-values combination test(AGCP) by optimizing the significant evidence of GCP obtained on each pair of a set of candidate thresholds applied to two sample location problem for arbitrary dimensional data since it is only based on marginal test statistics and poses no demands on the dimensionality. Extensive simulations show that the proposed test perform more powerful than some existing methods for two-sample location problem in high-dimensional, while maintaining correct type I error rates. The superiority of the proposed method is further exemplified with the Ageing Human Brain miacroarray data. In the analysis of this data, the proposed method succeeds in detecting the significant difference while other methods failed to do so.

Suppose that there are two independently and identically distributed random samples as follows: ${X}_{11},{X}_{12},\cdots ,{X}_{1{n}_{1}}\sim {F}_{1}(X-{\mu }_{1})$, ${X}_{21},{X}_{22},\cdots ,{X}_{2{n}_{2}}\sim {F}_{2}(X-{\mu }_{2})$, where ~*F*_*i*_ is a distribution function in ${ {\mathcal R} }^{m}$ located at *m*-variate center ${\mu }_{i}={({\mu }_{i1},{\mu }_{i2},\cdots ,{\mu }_{im})}^{\tau }$ and ${X}_{ij}={({X}_{ij1},{X}_{ij2},\cdots ,{X}_{ijm})}^{\tau }$, $j=\mathrm{1,}\,\mathrm{2,}\,\cdots ,\,{n}_{i}$, *i* = 1, 2. Let *n* = *n*_1_+*n*_2_. The hypothesis of interest is

1 $${H}_{0}:{\mu }_{1}={\mu }_{2}\,{\rm{versus}}\,{H}_{1}:{\mu }_{1}\ne {\mu }_{2}.$$

## Results

### Simulation Results

In this section, we investigate the performance of the proposed test via simulation studies in terms of type I error rate and power for high-dimensional data in comparison with Chen and Qin’s test(abbreviated as CQ)^4^, Srivastava *et al*.’s test (SKK)^5^, and Feng *et al*.’s test (SS)^6^. For a more general illustration, the AGCP test used here is assembled with the two-sample wilcoxon test for each marginal hypothesis. Three simulation models including multivariate normal distribution, multivariate t-distribution, and moving average model are considered to generate two-sample data. The specific scenarios are as follows: the first one is for multivariate normal distribution (MVN). ${X}_{ij}\sim N({\mu }_{i},{{\rm{\Sigma }}}_{i})$, *i* = 1, 2, $j=\mathrm{1,}\,\mathrm{2,}\,\cdots ,\,{n}_{i}$; the second one is for multivariate t-distribution *t*_*m*,4_(MVT). *X*_*ij*_ are sampled from *t*_*m*,4_ with 4 degrees of freedom, the mean vector *μ*_*i*_, and covariance matrix Σ_*i*_, *i* = 1, 2, $j=\mathrm{1,}\,\mathrm{2,}\,\cdots ,\,{n}_{i}$; the third one is for moving average model (MA). The *k*-th entry of *X*_*ij*_ are sampled from the following moving average structure: ${X}_{ijk}={\rho }_{1}{Z}_{ijk}+{\rho }_{2}{Z}_{ijk+1}+\cdots +{\rho }_{m}{Z}_{ijk+m-1}+{\mu }_{ik}$ for $i=\mathrm{1,}\,\mathrm{2,}\,j=\mathrm{1,}\,\mathrm{2,}\,\cdots ,\,{n}_{i}$ and $k=\mathrm{1,}\,2\cdots ,\,m$, and *Z*_*ijk*_ are i.i.d random variables. For the distribution of *Z*_*ijk*_, we let the first *m*/2 components of ${\{{Z}_{ijk}\}}_{k=1}^{m}$ be from centralized Gamma (4, 1) so that it has zero mean, and the other *m*/2 components from the standard normal distribution *N*(0, 1). Detailed settings of the other parameters will be introduced later.

Without loss of generality, we fix *μ*_2_ = 0 and ${{\rm{\Sigma }}}_{2}={I}_{m\times m}$ throughout the simulations, where ${I}_{m\times m}$ is an *m* × *m* identity matrix. Moreover, we assumed that *n*_1_ = *n*_2_ = *n*, taking values from {10, 25, 50}. For each sample size, the dimension was set to be 100 or 200. This leads to six combinations of (*n*, *m*): (*n*, *m*) = (10, 100), (10, 200), (25, 100), (25, 200), (50, 100), and (50, 200). For the covariance matrix ${{\rm{\Sigma }}}_{1}={({\sigma }_{u,v})}_{m\times m}$, $u,v=\mathrm{1,}\,2\cdots ,m$, we considered three dependence structures: (1) uniform moderate covariances with equal variance: ${\sigma }_{u,u}=1$, ${\sigma }_{u,v}=0.5$ when *u* ≠ *v* (denoted by DS1); (2) a gradient of moderate to low covariances with equal variance: ${\sigma }_{u,v}={0.5}^{|u-v|}$ (denoted by DS2); (3) a gradient of moderate to low covariances with different variances: ${\sigma }_{u,u}=1$, when $u\in \mathrm{\{1},\,\cdots ,m\mathrm{/2\}}$, ${\sigma }_{u,u}=3$ when $u\in \{m\mathrm{/2}+\mathrm{1,}\,\cdots ,m$}, and ${\sigma }_{u,v}={0.5}^{|u-v|}$ when *u* ≠ *v*, where $u,v=\mathrm{1,}\,\mathrm{2,}\,\cdots ,\,m$ (denoted by DS3).

To assess the performance of the tests on controlling type I error rates, we set *μ*_1_ = *μ*_2_. Clearly the null hypothesis (1) is true under this setting. In addition, under the alternative hypothesis, we let ${\mu }_{1}={({\mu }_{11},{\mu }_{12},\cdots ,{\mu }_{1m})}^{\tau }$ possess *L* non-zero entries, where *L* > 0. For a meaningful power comparison, different levels of significance were considered by varying *L*. We set $L=\lfloor {m}^{\gamma }\rfloor$ and choose *γ* from {0.1, 0.2, 0.3, 0.4}, where $\lfloor x\rfloor$ denote the largest integer less than or equal to *x*. Similar to Chen and Qin^4^, we use two patterns of allocations for the nonzero entries *μ*_1*l*_, $l=\mathrm{1,}\,\mathrm{2,}\,\cdots ,\,L$. One is the equal allocation where all nonzero entries *μ*_1*l*_ are equal; the other is linear allocation where all the nonzero *μ*_1*l*_ are linearly increasing. To make the power comparable among all configurations, we set $\eta =||{\mu }_{1}-{\mu }_{2}{||}^{2}/\sqrt{{\rm{tr}}({{\rm{\Sigma }}}_{1}^{2})+{\rm{tr}}({{\rm{\Sigma }}}_{2}^{2})}$ and the specific value of *μ*_1_ was obtained from this relation. *η* was chosen to be 0.2 for two combinations with (*n*, *m*) = (10, 100), (10, 200), 0.1 for (*n*, *m*) = (25, 100), (50, 200), and *η* = 0.05 for (*n*, *m*) = (50, 100), (50, 200). All results are calculated based on 1,000 simulations and the nominal level *α* is set to be 0.05. To save space, here we only present the power results for (*n*, *m*) = (10, 100) and (*n*, *m*) = (25, 200); results for the other configurations of (*n*, *m*) are similar and presented in the Supplementary Materials.

All simulation results are calculated based on 1000 monte carlo replications and *B* = 10000 permutations are used to compute the inner marginal p-values.

### Multivariate normal distribution

Table 1 reports the empirical type I error rates of CQ, SKK, SS, and AGCP when the two-sample data are generated from *m*-variate normal distribution with three patterns of covariance matrix including DS1, DS2 and DS3. From this table, it can be seen that SS and AGCP maintain correct type I error rates with the values being close to the nominal level. When the sample size is small (*n* = 10), SKK yields inflated type I error rates. For example, when *n* = 10 and *m* = 10, the type I error rates of SKK under DS1, DS2 and DS3 are 0.084, 0.097 and 0.119, respectively. For CQ, it can control the type I error rates correctly in most cases, while appears to be a little larger than 0.05 when the sample size is small and the covariance matrix belongs to DS1.

**Table 1:** Type I error rates of CQ, SKK, SS, and AGCP under the significance level of 0.05 when the two-sample data are generated from multivariate normal distribution.

n		m = 100				m = 200			
		CQ	SKK	SS	AGCP	CQ	SKK	SS	AGCP
10	DS1	0.087	0.084	0.058	0.058	0.068	0.062	0.051	0.040
	DS2	0.043	0.097	0.009	0.047	0.045	0.118	0.003	0.048
	DS3	0.042	0.119	0.004	0.039	0.047	0.160	0.001	0.049
25	DS1	0.059	0.043	0.054	0.044	0.059	0.044	0.069	0.058
	DS2	0.042	0.044	0.032	0.037	0.058	0.057	0.041	0.048
	DS3	0.039	0.049	0.023	0.050	0.062	0.063	0.032	0.042
50	DS1	0.041	0.027	0.055	0.038	0.060	0.032	0.067	0.041
	DS2	0.040	0.038	0.043	0.052	0.032	0.024	0.025	0.047
	DS3	0.053	0.050	0.051	0.044	0.033	0.032	0.034	0.054

DS1-DS3 correspond to three patterns of dependence structures for Σ_1_, respectively. *n* is the sample size and *m* is the data dimension.

**Figure 1:**
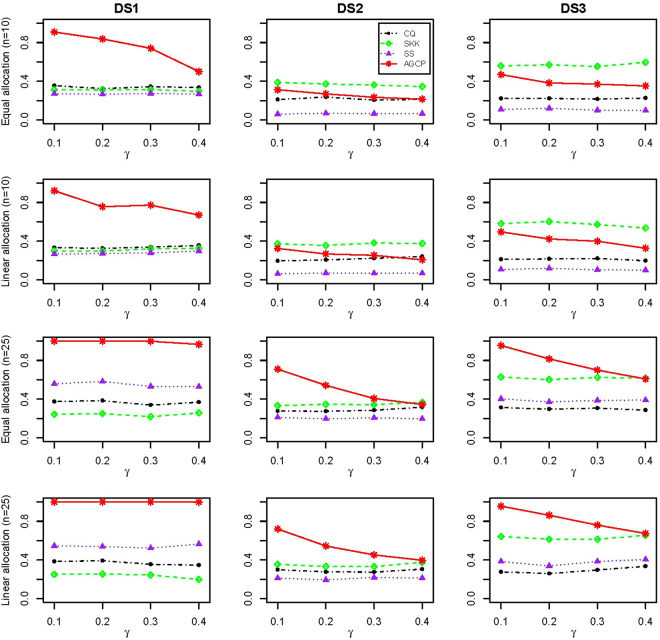
Empirical powers of CQ, SKK, SS, and AGCP for two-sample data generated from multivariate normal distribution with (*n*, *m*) = (10, 100) (Row 1 and 2) and (*n*, *m*) = (25, 200) (Row 3 and 4). For each combination of (*n*, *m*), two allocations (denoted by Equal and Linear allocation) are specified for the nonzeros of *μ*_1_. DS1-DS3 correspond to three patterns of dependence structures for Σ_1_, respectively.

The empirical powers of tests for the two-sample data sampled from multivariate normal data with (*n*, *m*) = (10, 100) and (*n*, *m*) = (25, 200) are presented in Fig. 1. Since SKK has inflated type error rates when the sample size is small, we excluded it from the power comparison when *n* = 10. Figure 1 shows that the powers of the proposed AGCP test are always larger than those of the other tests. Sometimes, its powers can exceed two times of those of the CQ, SKK and SS test. For example, when (*n*, *m*) = (10, 100) and *γ* = 0.1, the powers of CQ, SKK, SS, and AGCP are 0.354, 0.313, 0.270, and 0.909, respectively. And the performance of all tests are similar under the equal allocation and linear allocation. For the covariance matrix, three patterns structures including DS1, DS2 and DS3 were considered. Under the structures of DS1 and DS3, the superiority of the proposed test is very significant over the other tests in terms of powers. Under DS2, CQ has similar powers to AGCP when the percentage γ of non-zero entries of *μ*_1_ is large. Power results for (*n*, *m*) = (10, 200), (25, 100), (50, 100), and (50, 200) are similar and presented in the Supplementary Materials.

### Multivariate t-distribution

The empirical type I error rates of the compared tests for the two-sample data from *m*-variate t-distribution are presented in Table 2. Among all settings, AGCP always can maintain the type I error rates correctly. Likewise, the type I error rates of CQ have some slight size distortion (a little larger than 0.05) when the sample size is 10 and the covariance structure is DS1. In other cases, they are close to the nominal significance level. For the multivariate t-distribution data, SKK has totally incorrect type I error rates. It occurs since SKK is exclusively designed for multivariate normal distribution. The performance of SS on type I error rate depend heavily on the covariance structure. Specifically, its type I error rates are a little larger than 0.05 under DS1 and appear to very low under DS2 and DS3, especially when the sample size is small (*n* = 0.3). This result is consistent with those in Feng *et al*.^6^.

**Table 2:** Type I error rates of CQ, SKK, SS, and AGCP under the significance level of 0.05 when the two-sample data are generated from multivariate t-distribution.

n		m = 100				m = 200			
		CQ	SKK	SS	AGCP	CQ	SKK	SS	AGCP
10	DS1	0.087	0.039	0.054	0.054	0.079	0.032	0.062	0.055
	DS2	0.045	0.003	0.003	0.057	0.052	0.000	0.002	0.036
	DS3	0.052	0.008	0.003	0.050	0.052	0.000	0.000	0.055
25	DS1	0.066	0.028	0.062	0.055	0.063	0.022	0.063	0.040
	DS2	0.047	0.004	0.021	0.046	0.055	0.000	0.025	0.054
	DS3	0.060	0.001	0.026	0.048	0.050	0.002	0.030	0.053
50	DS1	0.047	0.017	0.068	0.057	0.058	0.019	0.069	0.055
	DS2	0.032	0.004	0.033	0.056	0.056	0.004	0.042	0.050
	DS3	0.056	0.002	0.036	0.048	0.047	0.001	0.042	0.049

DS1-DS3 correspond to three patterns of dependence structures for Σ_1_, respectively. *n* is the sample size and *m* is the data dimension.

**Figure 2:**
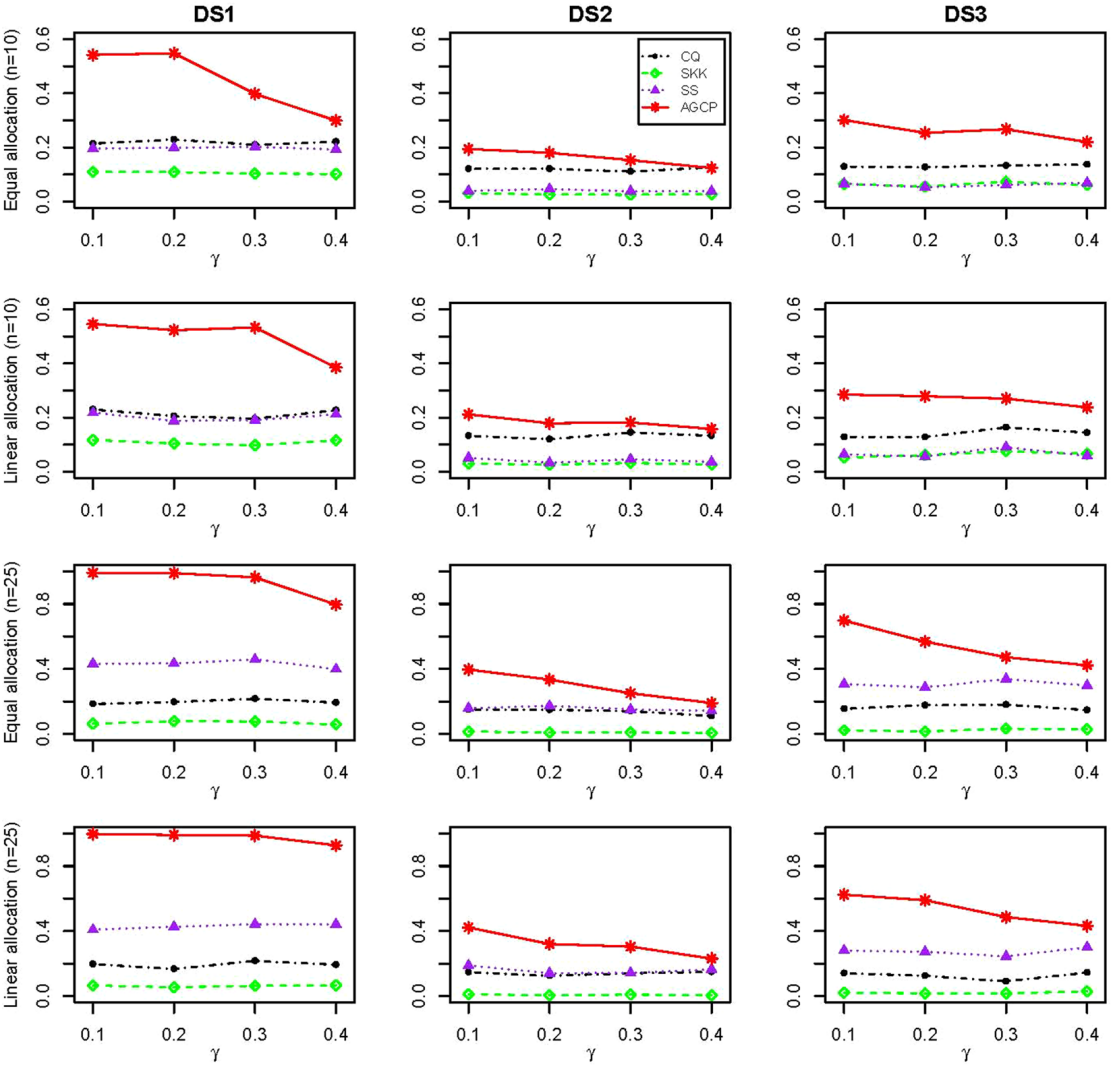
Empirical powers of CQ, SKK, SS, and AGCP for two-sample data generated from multivariate t-distribution with (*n*, *m*) = (10, 100) (Row 1 and 2) and (*n*, *m*) = (25, 200) (Row 3 and 4). For each combination of (*n*, *m*), two allocations (denoted by Equal and Linear allocation) are specified for the nonzeros of *μ*_1_. DS1-DS3 correspond to three patterns of dependence structures for Σ_1_, respectively.

Figure 2 shows the empirical powers of CQ, SKK, SS, and AGCP for two-sample data generated from multivariate t-distribution with the covariance structures of DS1, DS2, and DS3. It can be clearly observed from this figure that AGCP is the most powerful test among all compared test under all considered cases. As the sample size increases, the superiority of AGCP over the other tests becomes large. For example, under DS1 with equal allocation and *γ* = 0.3, the powers of CQ, SKK, SS, and AGCP for (*n*, *m*) = (10, 100) are 0.210 0.103, 0.202, and 0.398, respectively, while their powers for (*n*, *m*) = (25, 200) are 0.216 (CQ), 0.076 (SKK), 0.458 (SS), and 0.964 (AGCP). Moreover, the results of tests under equal allocation and linear allocation are similar. The power results for (*n*, *m*)=(10, 200), (25, 100), (50, 100) and (50, 200) are available in the Supplementary Materials.

### Moving average model

In addition, we examined the performance of tests for the data from moving average models. We choose a representative distribution for *Z*_*ijk*_, that is, letting the first *m*/2 components ${Z}_{ij1},{Z}_{ij2},\cdots ,{Z}_{ij\frac{m}{2}}$ sampled from Gamma (4, 1) − 4 and the other *m*/2 components from *N* (0, 1), $i=1,2$, $j=\mathrm{1,}\,\mathrm{2,}\,\cdots ,{n}_{i},\,k=\mathrm{1,}\,2\cdots ,m$. Note that both distributions have zero means. The correlations among *X*_*ij*_ are determined by the coefficients $\rho ={({\rho }_{1},{\rho }_{2},\cdots ,{\rho }_{m})}^{\tau }$. Two configurations of dependence structure for ${X}_{ij},i=\mathrm{1,}\,\mathrm{2,}\,j=\mathrm{1,}\,\mathrm{2,}\,\cdots ,\,{n}_{i}$, are considered. They are “full dependence” case with all the coefficients ${\rho }_{l},l=\mathrm{1,}\,2\cdots ,m$, are nonzero and “partial dependence” case with ${\rho }_{l}=0$ if *l* > 3 which means that ${X}_{ij{k}_{1}}$ and ${X}_{ij{k}_{2}}$ are dependent only if $|{k}_{1}-{k}_{2}| < 3$. Similar to Chen and Qin^4^, we generate independently the non-zero *ρ*_*l*_ from the uniform distribution *U* (2, 3) and kept them fixed throughout the simulation. With the values of *ρ*_*l*_, together with the relation $\eta =||{\mu }_{1}-{\mu }_{2}{||}^{2}/\sqrt{{\rm{tr}}({{\rm{\Sigma }}}_{1}^{2})+{\rm{tr}}({{\rm{\Sigma }}}_{2}^{2})}$, the specific setting for *μ*_1_ can be subsequently obtained.

**Figure 3:**
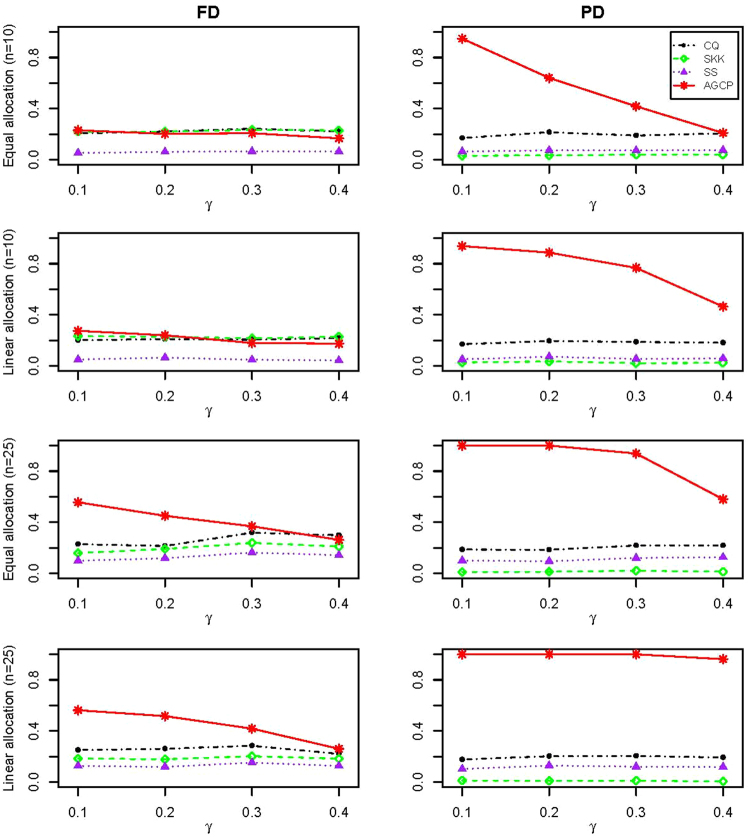
Empirical powers of CQ, SKK, SS, and AGCP for two-sample data generated from moving average model (*n*, *m*) = (10, 100) (Row 1 and 2) and (*n*, *m*) = (25, 200) (Row 3 and 4). For each combination of (*n*, *m*), two allocations (denoted by Equal and Linear allocation) are specified for the nonzeros of *μ*_1_ Two configurations including “full dependence“and “partial dependence” (denoted by FD and PD).

Table 3 summarizes the type I error rates of CQ, SKK, SS, and AGCP for the data from moving average models. It shows that CQ and AGCP maintain the type I error rates reasonably well, while SKK and SS seem to be somewhat conservative with the type I error rates much smaller than the nominal significance level. Similar to the results for MVN and MVT, the type I error rates of SKK when *n* = 10 is a little bit inflated. The powers of tests are presented in Fig. 3. From this figure, we can observe that the proposed test always performs the best among all tests when the coefficients are with the “partial dependence” structure for both (*n*, *m*) = (10, 100) and (*n*, *m*) = (25, 200). And such superiority becomes more significant as *n* increases. Under the “full dependence” structure, CQ, SKK, and AGCP perform similarly when *n* = 10, but AGCP outperforms the other two when the sample size becomes large.

**Table 3:** Type I error rates of CQ, SKK, SS, and AGCP under the significance level of 0.05 when the two-sample data are generated from moving average model (Replicate 200 times).

n		m = 100				m = 200			
		CQ	SKK	SS	AGCP	CQ	SKK	SS	AGCP
10	FD	0.051	0.069	0.010	0.051	0.041	0.092	0.005	0.054
	PD	0.061	0.026	0.038	0.043	0.059	0.012	0.029	0.043
25	FD	0.043	0.036	0.027	0.037	0.044	0.041	0.032	0.050
	PD	0.041	0.007	0.038	0.036	0.059	0.003	0.043	0.045
50	FD	0.048	0.039	0.051	0.053	0.046	0.038	0.049	0.052
	PD	0.067	0.012	0.064	0.047	0.058	0.007	0.041	0.045

Two configurations including “full dependence” and “partial dependence” (denoted by FD and PD) are used to generate the coefficients *ρ*_*l*_, $l=\mathrm{1,}\,\mathrm{2,}\,\cdots ,m$. *n* is the sample size and *m* is the data dimension.

The type I error rates seems to be slightly inflated when the sample size is 10. This occurs because under such situation, the sample size is too small relative to the dimension of data which maybe need more permutation replications to approximate p-values of marginal tests in AGCP.

### Applications to Ageing Human Brain Microarray data

To further exemplify the superiority of the proposed test, we apply it to the Ageing Human Brain Microarray (AHBM) data, downloaded from GEO with accession number GSE1572 (https://www.ncbi.nlm.nih.gov/geo/query/acc.cgi?acc=GSE1572). Ageing of human brain, accompanied by slower processing speeds and decreasing ability to convert experiences to episodic memory, is known as a cause of cognitive decline and potentially risk factors of age-related neurodegenerative diseases, such as Alzheimer’s disease^13–15^. The AHBM data were used by Lu *et al*.^16^ to detect age-dependent gene regulation in human brain. It contains microarray expression patterns of genes from the frontal cortex of 30 neuropathologically normal individuals ranging in age from 26 to 106 years. Lu *et al*.^16^ showed that the gene expression patterns are relatively stable among the group of individuals ≤42 years old. In addition, they performed statistical group comparison of expression levels from individuals ≤42 and ≤42 years old, about 4% of all genes were detected to undergo significant changes. Most of these genes were found to be associated with some functions, such as synaptic function, stem cell function, vesicular/protein transport, stress response, and others. Among the detected genes, we choose those belonged to some aging-associated pathways to form a gene set in the later analysis.

Five pathways including hedgehog signaling, mitogen-activated protein kinases/extracellular signal-regulated kinases (MAPK/ERK), phosphatidy linositol 3-kinase (PI3K), protein kinase C (PKC), and janus kinases/signal transducers and activator of transcription (JAK/STAT) which are reported to be aging-associated, were chosen. Specifically, hedgehog signaling is a major regulator of stem cell function whose reduced functionality is responsible for ageing^17^; the core components or regulators of MAPK/ERK pathway were identified as the aging-dependent targets^18^; the PI3K pathway was found to have relevance to cognitive processes in addition to pathological brain aging and neuro degeneration since it is implicated in aging and lifespan regulation, and the proliferation of adult neuronal progenitor cells^19^; the PKC pathway and its adaptor protein RACK1 have been shown to be interdependent in pathological brain aging^20^; JAK/STAT were found to be active in the aging and mature brain and play important role in the control of neuronal proliferation, survival and differentiation^21^. The pathway data was downloaded from InnateDB http://www.innatedb.ca/redirect.do?go=searchPws.

A total of 237 genes (listed in the Supplementary Materials) were included in the gene set. Since the individuals ≤42 years old in this data share similar gene expression patterns, all individuals are divided into two sample groups consisting of 10 individuals ≤42 years old and 20 individuals ≥42 years old in the analysis, respectively. Our aim is to detect simultaneously the difference of expression patterns of the gene set between two samples. To provide some insights into each gene’s expression pattern, univariate comparison between two groups were first conducted using the wilcoxon rank-sum test and the p-values ranges from 0.00012 to 1. The detailed marginal p-values are presented in the Supplementary Materials. Then we apply the tests of Chen and Qin^4^, Srivastava *et al*.^5^, Feng *et al*.^6^, and the proposed test to conduct the overall comparison of expression patterns of the gene set between two groups. The p-values are 0.221 from Chen and Qin’s test^4^, 0.089 from Srivastava *et al*.’s test^5^, 0.051 from Feng *et al*.’s test, and 0.017 from the proposed new test, indicating that only the proposed test succeeded in detecting the difference of expression patterns between two groups.

## Discussion

Through simulation studies, we show that the proposed test outperforms some competing multivariate tests with respect to the type I error rate and power in most scenarios. This is expected since the compared tests including CQ, SKK, and SS which are all Hotelling’s *T*^2^-type tests, neglect the correlations among variables to bypass the non-convergence of the sample covariance matrix (Bai and Saranadasa, 1996), while our method takes the correlation of multiple variables into account and calculate the statistical significance level with the permutation method.

In this article, we developed an adaptive group-combined p-values procedures for two-sample location problem in high-dimensional data. The proposed test extends the p-value combining techniques by dividing p-values into several groups and combing them at the group-level. Instead of fixed thresholds, this adaptive procedure use the optimal one among all possible threholds which is able to improve the power of test significantly. The proposed test provides an efficient and flexible way to accumulate differece evidences across variables and has no restriction on the relationship between the data dimension and sample size. Through simulation studies, we showed that the proposed test outperformed some competing multivariate tests in most scenarios. Applications to Ageing Human Brain Microarray data further demonstrate its satisfactory performance.

In the proposed test, all p-values are divided into three groups and two groups with smaller p-values are used. However, the number of groups is sort of self-defined. Intuitively, such procedure can be generalized to *J* groups, *J* ≥ 3. Although Hu *et al*.^12^ explained *J* = 3 is a good choice through simulation studies and the idea of the degrees of freedom, more theoretical results are needed to support this conclusion. Except the two-sample location problem, our proposed test have a variety of additional applications, such as large-scale genetic association studies. With the advance of high-throughput genotyping technology, researchers are able to get access to a large number of genetic variants. However, the signal of association between an individual genetic variant and the trait could be too weak to be detected by single-variant analysis^22,23^. At this time, a benefiting and complementary strategy for genetic association studies is to simultaneously testing the association between the trait and multiple genetic variants within a gene set or a pathway. A specific high-dimensional test problem thus arises. Our proposed method can be applied by conducting marginal association test for each genetic variant first and then use the proposed test to combine obtained p-values. Our method can also be extended to deal with nonparametric population comparisons in genetic association studies, where much work has been done^24–26^.

For our proposed methods, we recommend using the thresholds $\xi =\mathrm{\{0.0001,}\,\mathrm{0.001,}\,\mathrm{0.01,}\,\mathrm{0.05,}\,\mathrm{0.1,}\,\mathrm{0.2,}\,\mathrm{1\}}$. This is mainly due to the following reasons. First, these thresholds are widely used in the context of p-values combination methods (Fisher^8^; Zaykin *et al*.^9^; Jiang *et al*.^11^; Yu *et al*.^11^). Besides, since the p-values greater than 0.2 generally do not contribute to the significance of test but may increase the variance substantially, the value of 0.2 is commonly used as the upper bound of threshold for the truncated p-value combination methods (Zaykin *et al*.^9^). Finally, we also evaluate the performance of the proposed test under some other sets of thresholds containing *ξ* through simulations and the results turn out to be similar with those in the “simulation” section; we omit the details here for simplicity.

Intuitively, such procedure can be generalized to *J* ≥ 3 groups. As Hu *et al*.^12^ pointed out the AGCP with *J* = 3 possesses the potential to outperform that that with *J* > 3 due to the idea of pseudo degrees of freedom (DFs) for test statistics. That is, since the test with the form of −2 ln(*X*) is known to follow from the Chi-squared distribution, the pseudo DFs of the AGCP with *J* = 3 and *J* = 4 are 4 and 6, respectively. As the number of groups increases, the DFs might increase which yields less powerful tests.

It should be pointed out that our test has its drawback. In principal, the proposed test is supposed to be applied to any dimensional data since it is based on marginal p-values. However due to the difficulty of deriving the exact distribution, permutation procedure is adopted to calculate the statistical significance of the proposed AGCP which may suffer intensive computation or even be infeasible when the data dimension is very large.

## Methods

In particular, the null hypothesis (1) is a global null hypothesis including *m* correlated marginal hypothesises in terms of the location parameter of each variable, that is, ${H}_{0}:{\mu }_{1k}={\mu }_{2k}\mathrm{,\ }k=\mathrm{1,}\,\mathrm{2,}\,\cdots ,m$. For each variable, we can use a certain test statistic, such as two sample t-test and Wilcoxon test, to test for the equality of location parameters in two samples and denote the obtained p-values by ${p}_{1},{p}_{2},\cdots ,{p}_{m}$. Let $\xi =\{{\xi }_{1},{\xi }_{2},\cdots ,{\xi }_{S}\mathrm{;\ 0} < {\xi }_{s} < \mathrm{1,}\,s=\mathrm{1,}\,\mathrm{2,}\,\cdots ,S\}$ be a set of *S* thresholds. Without loss of generality, we assume that ${\xi }_{1}\le {\xi }_{2}\le \cdots \le {\xi }_{S}$. For each pair of thresholds ${\xi }_{{s}_{1}}$ and ${\xi }_{{s}_{2}}$, the group-combined p-values test statistic is given by

2 $$\begin{array}{rcl}{\rm{GCP}}({\xi }_{{s}_{1}},{\xi }_{{s}_{2}}) & = & -2\,\mathrm{ln}\{1-{F}_{{s}_{1}}(-2\sum _{k=1}^{m}\,\mathrm{ln}\,[{p}_{k}]I({p}_{k} < {\xi }_{{s}_{1}}))\}\\  &  & -2\,\mathrm{ln}\{1-{F}_{{s}_{2}}(-2\sum _{k\mathrm{=1}}^{m}\,\mathrm{ln}\,[{p}_{k}]I({\xi }_{{s}_{1}}\le {p}_{k} < {\xi }_{{s}_{2}}))\},\end{array}$$

where ${F}_{{s}_{1}}$ and ${F}_{{s}_{2}}$ are the cumulative distribution functions of $-2\sum _{k=1}^{m}\,\mathrm{ln}\,[{p}_{k}]I({p}_{k} < {\xi }_{{s}_{1}})$ and $-2\sum _{k\mathrm{=1}}^{m}\,\mathrm{ln}\,[{p}_{k}]I\times$ $({\xi }_{{s}_{1}} < {p}_{k} < {\xi }_{{s}_{2}})$ for ${s}_{1},{s}_{2}\in \mathrm{\{1,}\,\mathrm{2,}\,\cdots ,S\}$, respectively. With this, the proposed AGCP test is presented as

3 $${\rm{AGCP}}=\mathop{{\rm{\max }}}\limits_{{s}_{1},{s}_{2}\in \mathrm{\{1,2,}\cdots ,S\}}{\rm{GCP}}({\xi }_{{s}_{1}},{\xi }_{{s}_{2}}).$$

It is worth pointing out that the selection of marginal tests is flexible due to that the proposed test is built on marginal p-values. Hence, we can choose an appropriate test solely for each single variable and these tests are not necessary to be the same. This, to some extent, improves the applicability of the proposed test. Moreover, AGCP imposes no restriction on the relationship between the data dimension and sample size since only marginal tests are conducted. In principal, it is able to handle any dimensional case. AGCP is an adaptive procedure which is expected to perform better with more thresholds. However, too many thresholds are not useful in the testing since they will lead to a lot of multiple comparisons. To limit the effect of multiple comparisons, we recommend using $\xi =\mathrm{\{0.0001,0.001,0.01,0.05,0.1,0.2,1\}}$ in the following sections which results in 21 pairs of thresholds. Note that if two thresholds (i.e., S = 2) are used, the AGCP test becomes the standard GCP test.

To calculate statistical significance of AGCP, we use a permutation procedure with taking the correlations among variables into account. Generally in our case, a two-layer permutation procedure is needed: the inner layer is used to calculate ${F}_{{s}_{1}},{F}_{{s}_{2}}$ and the outer layer is for the adjustment accounted for multiple tests over different pairs of thresholds. However, such two-layer permutation procedure is computationally intensive, especially when the number of thresholds is large. As an alternative, we proposed the following one-layer permutation algorithm to compute the p-value of AGCP:

### Algorithm

Step 1. Conduct marginal tests for each data dimension and denote the obtained p-values by ${p}_{1}^{\mathrm{(0)}},{p}_{2}^{\mathrm{(0)}},\cdots ,{p}_{m}^{\mathrm{(0)}}$
Step 2. Set a large number *B*, for example, *B* = 10000. For *b* from 1 to *B*, permutate the original observations $\{{X}_{11},{X}_{12},\cdots ,{X}_{1{n}_{1}},{X}_{21},{X}_{22},\cdots ,{X}_{2{n}_{2}}\}$ and denote the permutated samples as $\{{X}_{11}^{\ast },{X}_{12}^{\ast },\cdots ,{X}_{1{n}_{1}}^{\ast },\,{X}_{21}^{\ast },{X}_{22}^{\ast },\cdots ,{X}_{2{n}_{2}}^{\ast }\}$, calculate marginal p-values for the permutated samples ${\{{X}_{1j}^{\ast }\}}_{j=1}^{{n}_{1}}$, ${\{{X}_{2j}^{\ast }\}}_{j=1}^{{n}_{2}}$ and denote them by ${p}_{1}^{(b)},{p}_{2}^{(b)},\cdots ,{p}_{m}^{(b)}$.
Step 3. Specify a set of thresholds $\xi =\{{\xi }_{1},{\xi }_{2},\cdots ,{\xi }_{S}\mathrm{;\ 0} < {\xi }_{s} < \mathrm{1,}\,s=\mathrm{1,}\,\mathrm{2,}\,\cdots ,\,S\}$. Based on ${p}_{1}^{(b)},{p}_{2}^{(b)},\cdots ,{p}_{m}^{(b)}$, $b=\mathrm{1,}\,\mathrm{2,}\,\cdots ,B$ in Step 2, for each pair of thresholds ${\xi }_{{s}_{1}}$ and ${\xi }_{{s}_{2}}$, obtain the empirical cumulative distribution function corresponding to ${F}_{{s}_{1}}$ and ${F}_{{s}_{2}}$ and denoted them by ${\hat{F}}_{{s}_{1}}$ and ${\hat{F}}_{{s}_{2}}$, ${s}_{1},{s}_{2}\in \mathrm{\{1,}\,\mathrm{2,}\,\cdots ,S\}$;
Step 4. For *b* from 0 to *B*, calculate the corresponding AGCP test statistics using ${\hat{F}}_{{s}_{1}}$, ${\hat{F}}_{{s}_{2}}$ and ${p}_{1}^{(b)},{p}_{2}^{(b)},\cdots ,{p}_{m}^{(b)}$ and denote them by AGCP_*b*_;
Step 5. The p-value of AGCP is given by
  4 $${\rm{p}}-{\rm{value}}=\frac{\#\{{{\rm{AGCP}}}_{b}\ge {{\rm{AGCP}}}_{0}\,\mathrm{:\ }b=\mathrm{1,}\,\mathrm{2,}\,\cdots ,B\}}{B},$$
  where the symbol is an operator used to count the number of elements in a set.

In principal, a large value of *B* is preferred since it can yield accurate results of p-value. However, increasing value of *B* would result in extensive computational cost. To balance such tradeoff, we use *B* = 10000 in this article.

The source of program R code used to perform the simulations is available in the supplementary material.

## Electronic supplementary material

Supplementary Information
