# Supplementary material for: Adaptive Group-combined P-values Test for Two-sample Location Problem with Applications to Microarray Data
Source: Sci Rep. 2018 May 25;8:8117. doi: 10.1038/s41598-018-26409-1 (PMC5970252; doi:10.1038/s41598-018-26409-1)
Supplement: Supplementary file 1 — Supplementary Information [file 41598_2018_26409_MOESM1_ESM.pdf]

Supplementary Materials for

Adaptive Group-combined P-values Test for Two-sample Location Problem with Applications to Microarray Data

Shenghu Zhang[1], Jiayan Zhu[2], and Zhengbang Li[3,*]

[1]School of Mathematical Sciences, University of Chinese Academy of Sciences, Beijing, 100190, China

[2]School of Information and Communication, Wuhan College, Wuhan, 430212, China

[3] School of Mathematics and Statistics & Hubei Key Laboratory of Mathematical Sciences, Central China Normal University, Wuhan, 430079, China

[*] Corresponding author: lizhengbang@mail.ccnu.edu.cn

Contents

1. Additional simulation results for simulated data from multivariate normal distribution.

2. Additional simulation results for simulated data from multivariate t-distribution.

3. Additional simulation results for simulated data from moving average model.

4. P-values for univariate group comparison of gene expression patterns in the Ageing Human Brain data.

5. R code for statistical method AGCP.

1. Additional simulation results for simulated data from multivariate normal distribution.

Figure S1. Empirical powers of CQ, SKK, SS, and AGCP for two-sample data generated from multivariate normal distribution with $(n, m) = (10, 200)$. Two allocations (denoted by Equal and Linear allocation) are specified for the nonzeros of $\boldsymbol{\mu}_1$. DS1-DS3 correspond to three patterns of dependence structures for $\boldsymbol{\Sigma}_1$, respectively.

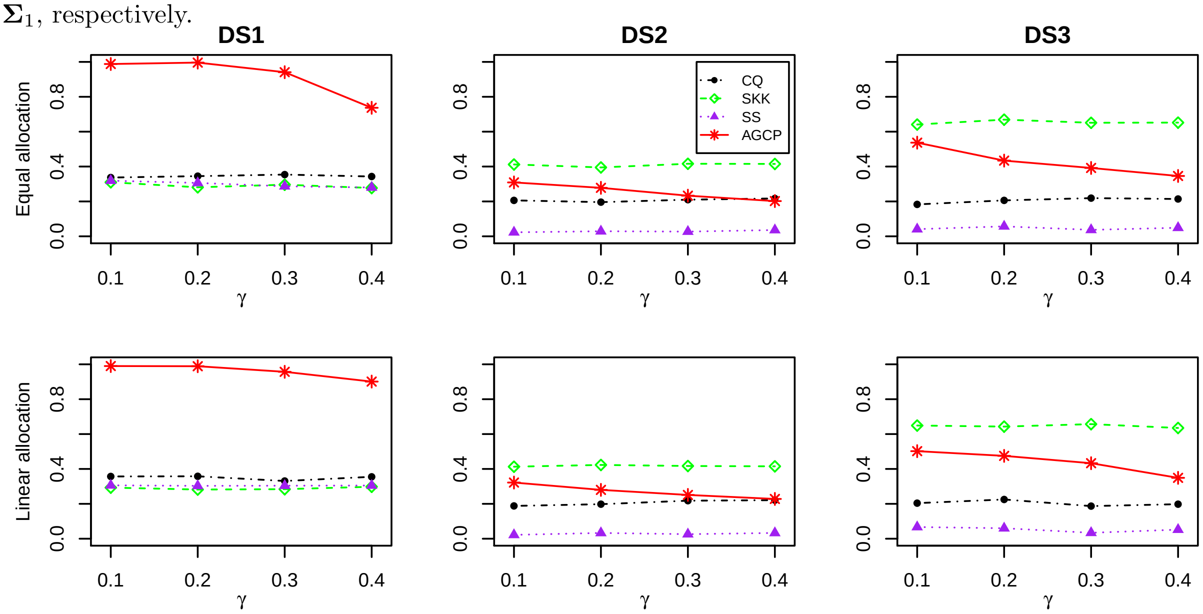

Figure S2. Empirical powers of CQ, SKK, SS, and AGCP for two-sample data generated from multivariate normal distribution with $(n, m) = (25, 100)$. Two allocations (denoted by Equal and Linear allocation) are specified for the nonzeros of $\boldsymbol{\mu}_1$. DS1-DS3 correspond to three patterns of dependence structures for $\boldsymbol{\Sigma}_1$, respectively.

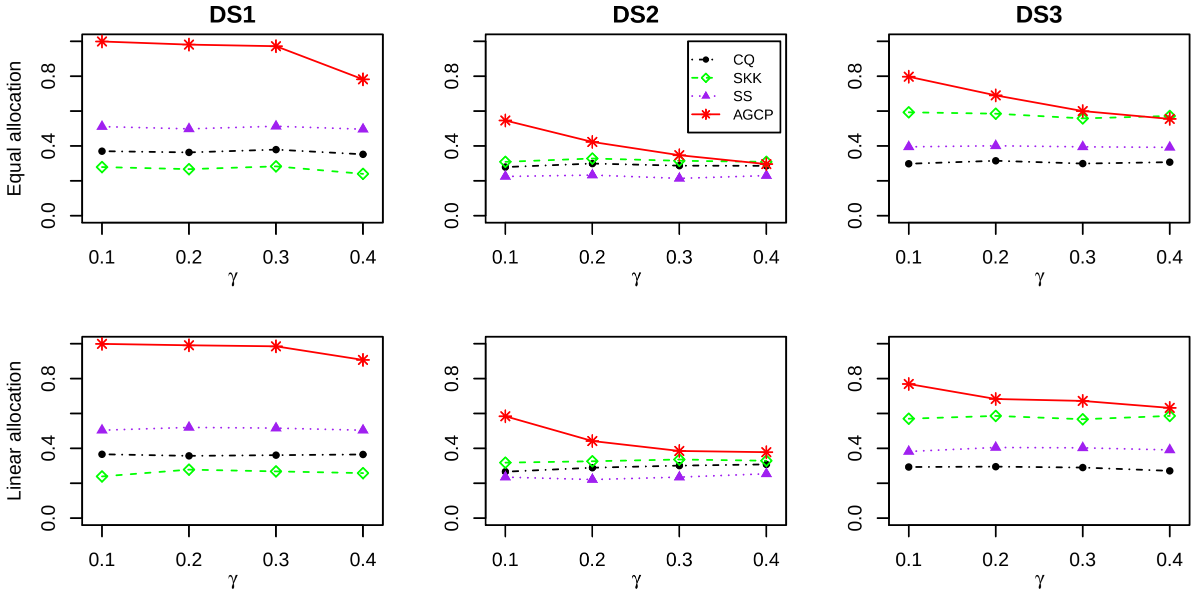

Figure S3. Empirical powers of CQ, SKK, SS, and AGCP for two-sample data generated from multivariate normal distribution with $(n, m) = (50, 100)$. Two allocations (denoted by Equal and Linear allocation) are specified for the nonzeros of $\boldsymbol{\mu}_1$. DS1-DS3 correspond to three patterns of dependence structures for $\boldsymbol{\Sigma}_1$, respectively.

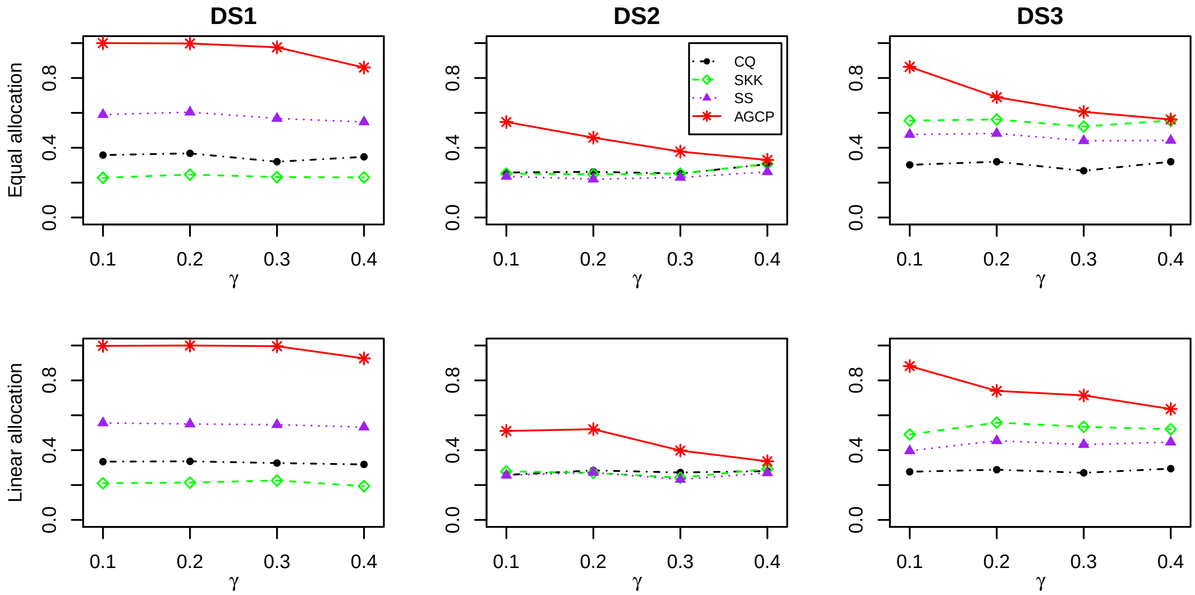

Figure S4. Empirical powers of CQ, SKK, SS, and AGCP for two-sample data generated from multivariate normal distribution with $(n, m) = (50, 200)$. Two allocations (denoted by Equal and Linear allocation) are specified for the nonzeros of $\boldsymbol{\mu}_1$. DS1-DS3 correspond to three patterns of dependence structures for $\boldsymbol{\Sigma}_1$, respectively.

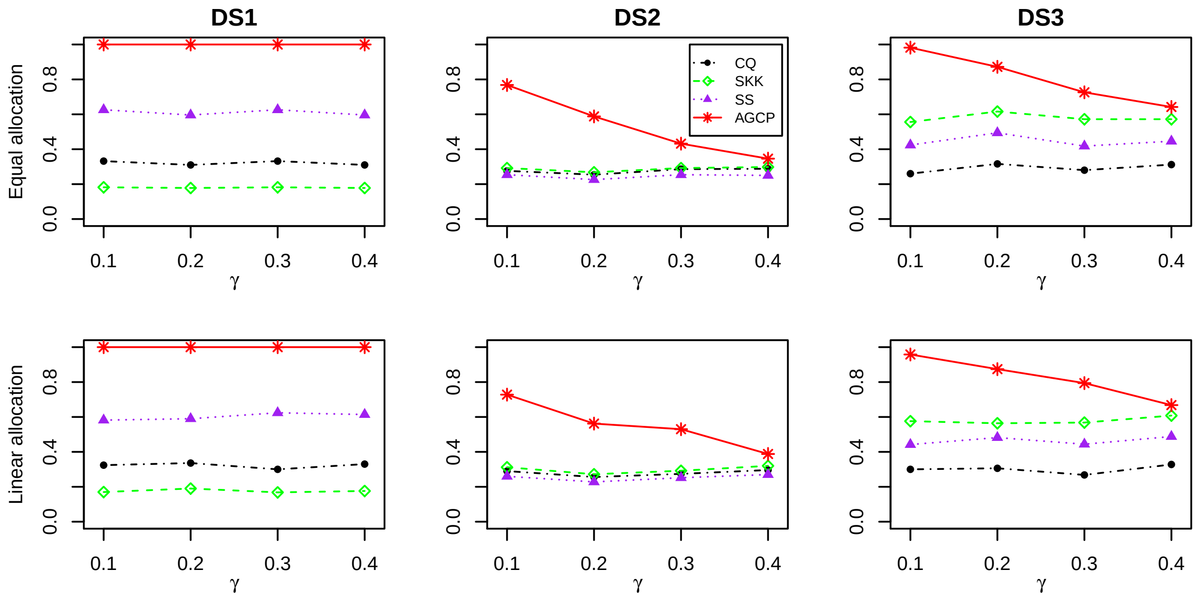

Table R1. Type I error rates of CQ, SKK, SS, and AGCP under the significance level of 0.05 when the two-sample data are generated from multivariate normal distribution with the sample size $n = 10$ and data dimension $m = 100$. DS1-DS3 correspond to three patterns of dependence structures for $\boldsymbol{\Sigma}_1$, respectively.

	CQ	SKK	SS	AGCP
DS1	0.092	0.098	0.072	0.056
DS2	0.060	0.114	0.010	0.043
DS3	0.042	0.110	0.002	0.045

Figure R1. Empirical powers of CQ, SKK, SS, and AGCP for two-sample data generated from multivariate normal distribution with $(n, m) = (10, 100)$. Two allocations (denoted by Equal and Linear allocation) are specified for the nonzeros of $\boldsymbol{\mu}_1$. DS1-DS3 correspond to three patterns of dependence structures for $\boldsymbol{\Sigma}_1$, respectively.

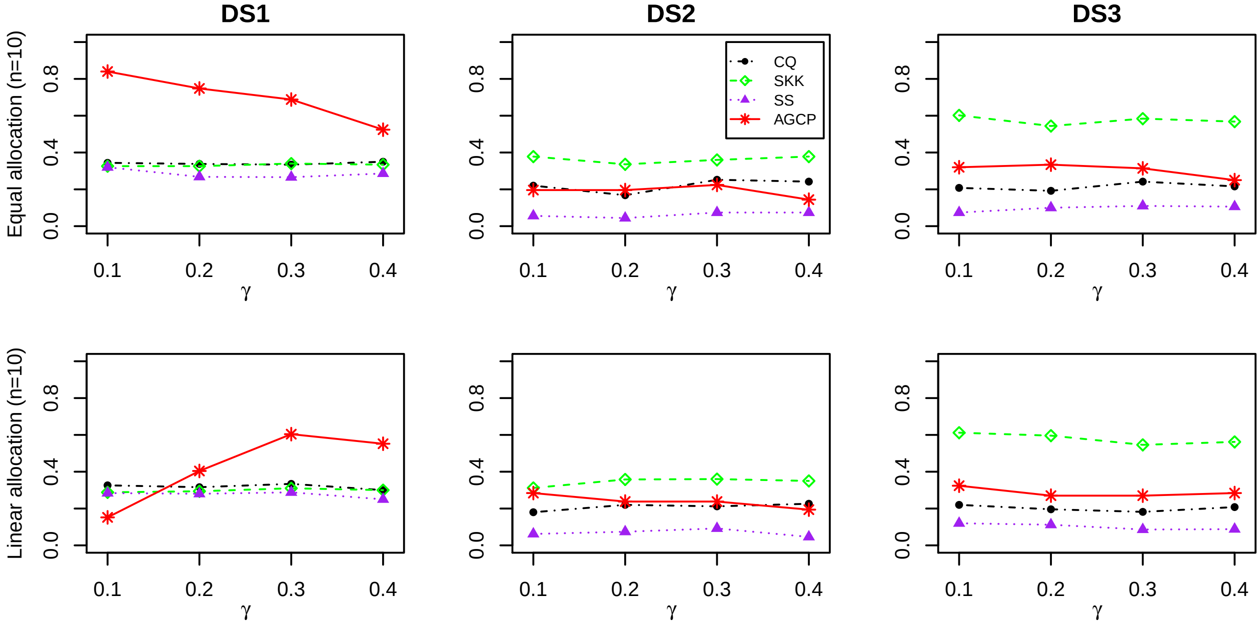

2. Additional simulation results for simulated data from multivariate-t distribution.

Figure S5. Empirical powers of CQ, SKK, SS, and AGCP for two-sample data generated from multivariate t-distribution with $(n, m) = (10, 200)$. Two allocations (denoted by Equal and Linear allocation) are specified for the nonzeros of $\boldsymbol{\mu}_1$ DS1-DS3 correspond to three patterns of dependence structures for $\boldsymbol{\Sigma}_1$, respectively.

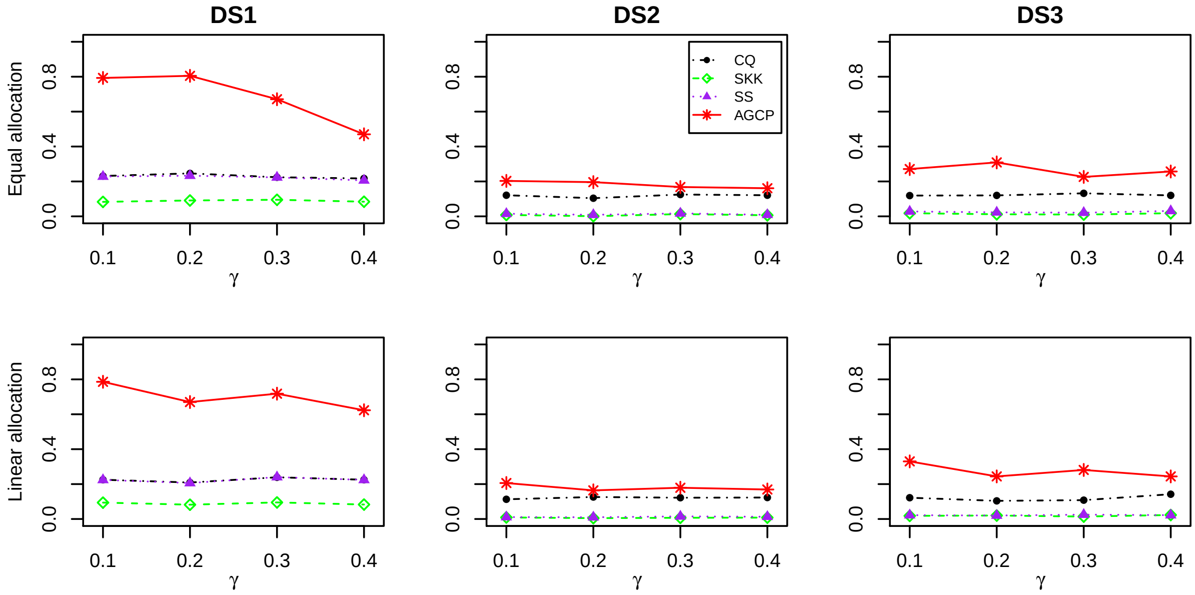

Figure S6. Empirical powers of CQ, SKK, SS, and AGCP for two-sample data generated from multivariate t-distribution with $(n, m) = (25, 100)$. Two allocations (denoted by Equal and Linear allocation) are specified for the nonzeros of $\boldsymbol{\mu}_1$. DS1-DS3 correspond to three patterns of dependence structures for $\boldsymbol{\Sigma}_1$, respectively.

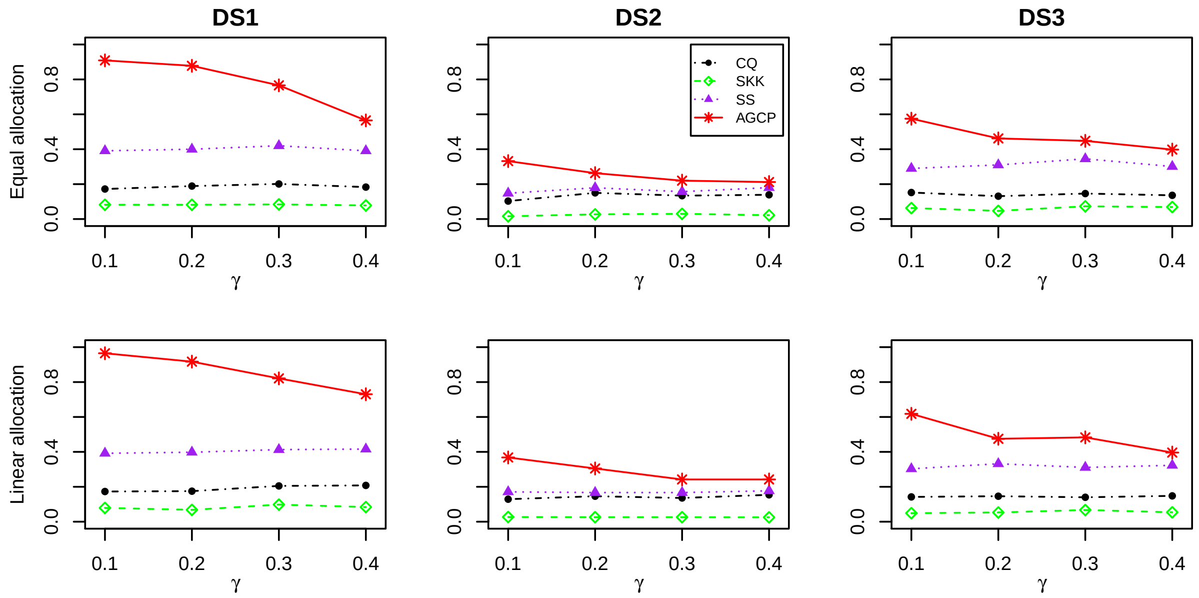

Figure S7. Empirical powers of CQ, SKK, SS, and AGCP for two-sample data generated from multivariate t-distribution with $(n, m) = (50, 100)$. Two allocations (denoted by Equal and Linear allocation) are specified for the nonzeros of $\boldsymbol{\mu}_1$. DS1-DS3 correspond to three patterns of dependence structures for $\boldsymbol{\Sigma}_1$, respectively.

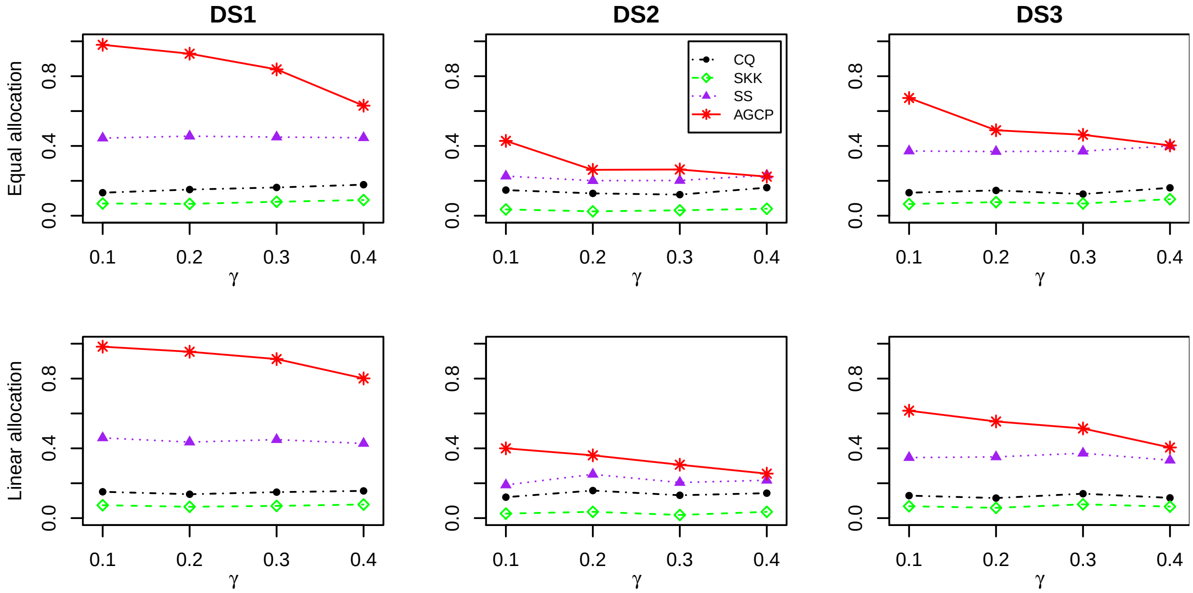

Figure S8. Empirical powers of CQ, SKK, SS, and AGCP for two-sample data generated from multivariate t-distribution with $(n, m) = (50, 200)$. Two allocations (denoted by Equal and Linear allocation) are specified for the nonzeros of $\boldsymbol{\mu}_1$. DS1-DS3 correspond to three patterns of dependence structures for $\boldsymbol{\Sigma}_1$, respectively.

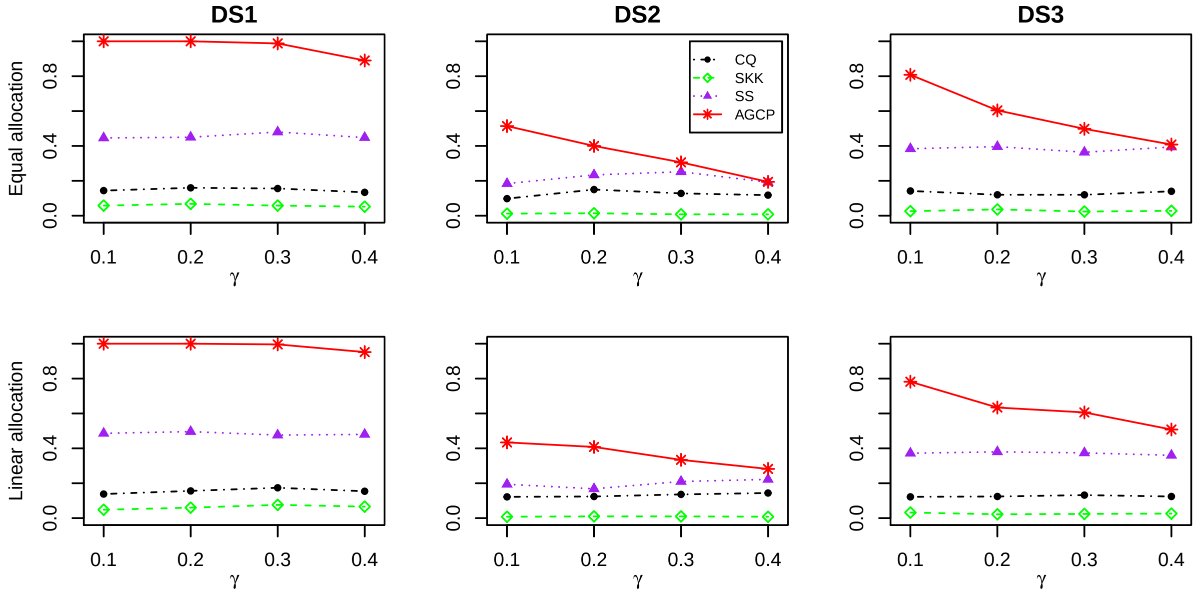

3. Additional simulation results for simulated data from moving average model.

Figure S9. Empirical powers of CQ, SKK, SS, and AGCP for two-sample data generated from moving average model with $(n, m) = (10, 200)$. Two allocations (denoted by Equal and Linear allocation) are specified for the nonzeros of $\boldsymbol{\mu}_1$. Two configurations called "full dependence" and "partial dependence" (denoted by FD and PD).

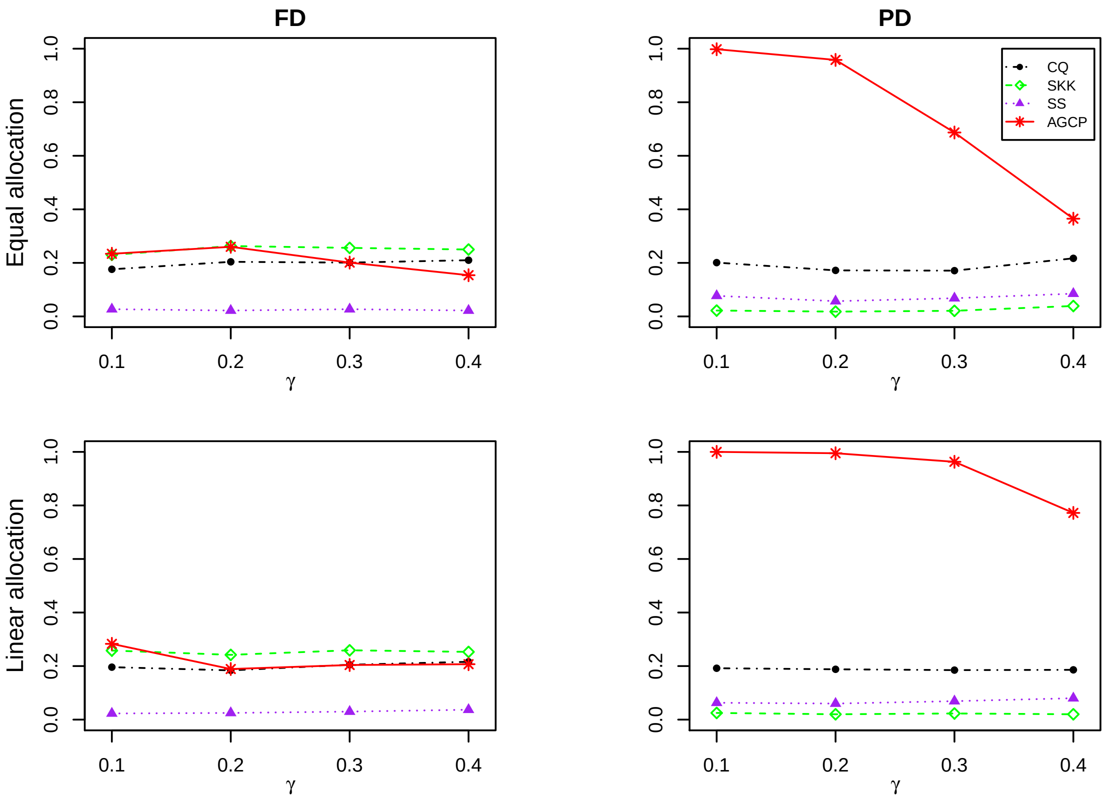

Figure S10. Empirical powers of CQ, SKK, SS, and AGCP for two-sample data generated from moving average model with $(n, m) = (25, 100)$. Two allocations (denoted by Equal and Linear allocation) are specified for the nonzeros of $\boldsymbol{\mu}_1$. Two configurations called "full dependence" and "partial dependence" (denoted by FD and PD).

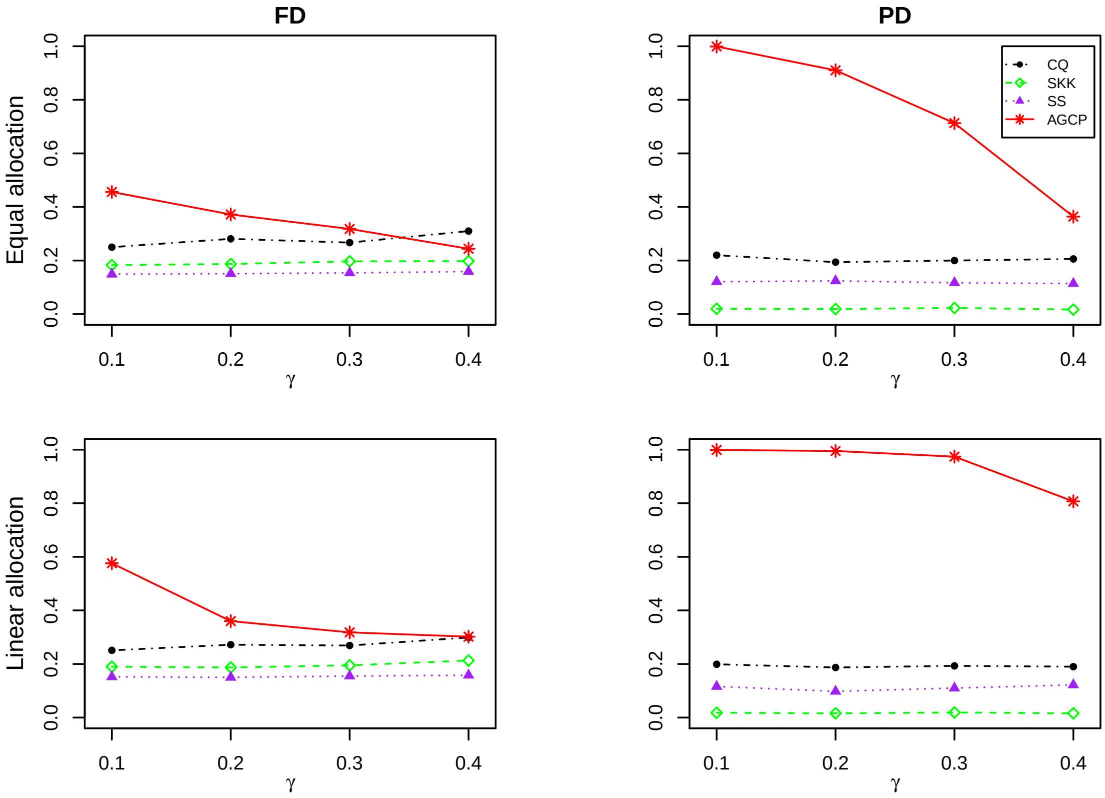

Figure S11. Empirical powers of CQ, SKK, SS, and AGCP for two-sample data generated from moving average model with $(n, m) = (50, 100)$. Two allocations (denoted by Equal and Linear allocation) are specified for the nonzeros of $\boldsymbol{\mu}_1$. Two configurations called "full dependence" and "partial dependence" (denoted by FD and PD).

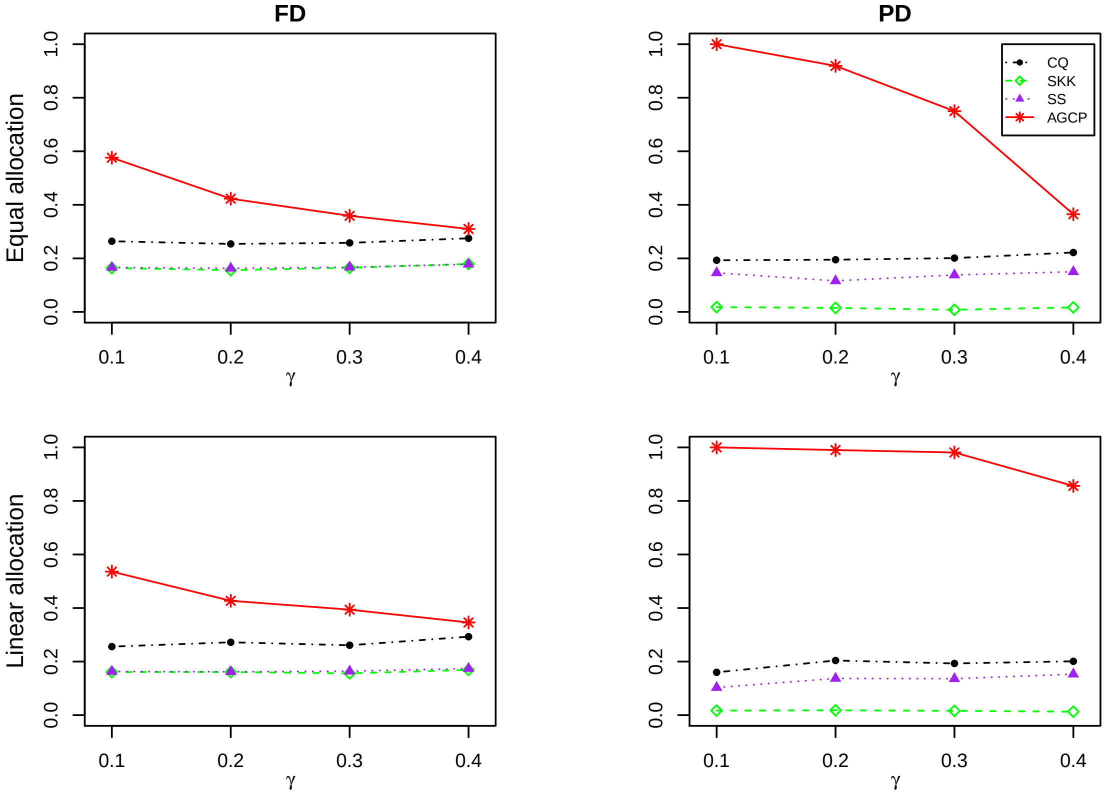

Figure S12. Empirical powers of CQ, SKK, SS, and AGCP for two-sample data generated from moving average model with $(n, m) = (50, 200)$. Two allocations (denoted by Equal and Linear allocation) are specified for the nonzeros of $\boldsymbol{\mu}_1$. Two configurations called “full dependence” and “partial dependence” (denoted by FD and PD).

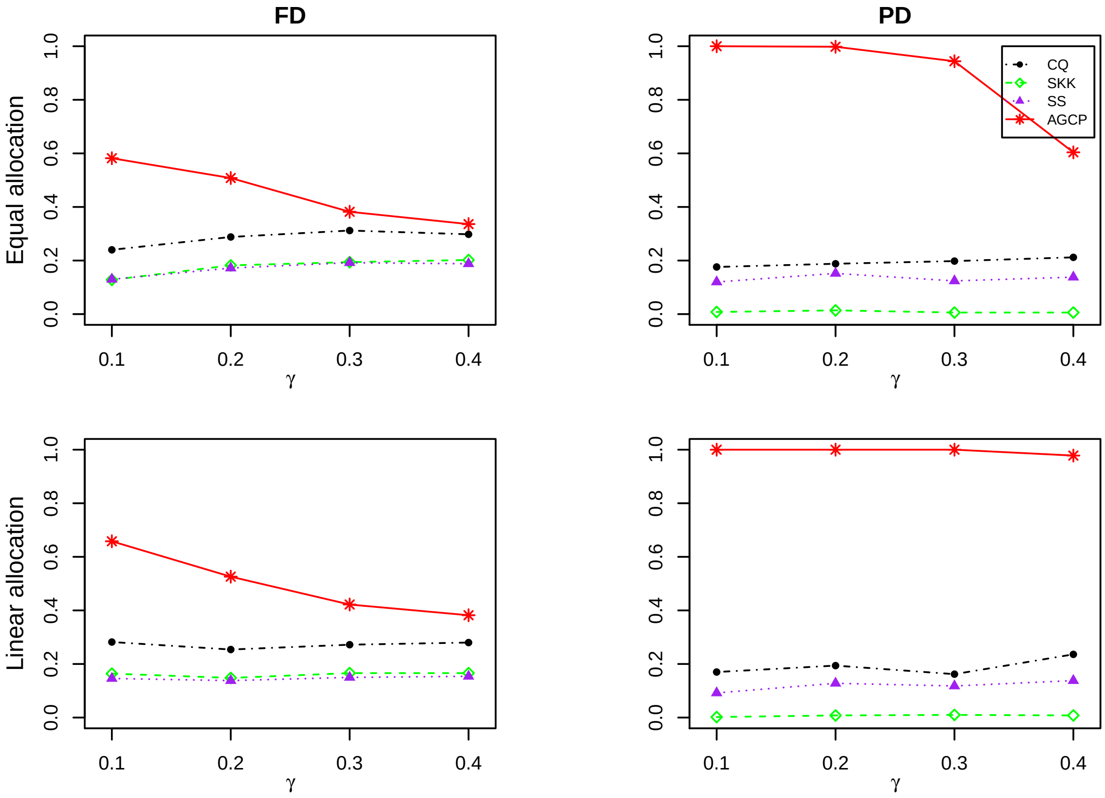

4. P-values for univariate group comparison of gene expression patterns in the Ageing Human Brain data.

Table S1. P-values for univariate comparison of expression patterns among 237 genes between two sample groups with individuals ≤ 42 and ≥ 42 years old.

geneID	p-value	geneID	p-value	geneID	p-value	geneID	p-value	geneID	p-value
BMP4	0.3442	PARP3	0.8431	XPA	0.6129	IFNAR1	0.3910	LEP	0.8088
GLI3	0.3442	LIG3	0.6441	RAD23B	0.3012	IFNGR2	0.0679	IL3RA	0.6760
WNT7A	0.7749	FEN1	0.3012	CUL4B	0.4414	PIAS2	0.2619	IL23A	0.3222
WNT10B	0.0615	PCNA	0.4157	CETN2	0.1291	LIF	0.8431	STAT2	0.1797
WNT1	0.9474	TDG	0.8088	RPA2	0.9474	OSM	0.0615	IL3	0.3671
DHH	0.0503	XRCC1	0.6760	GTF2H4	0.0679	SOS2	0.0557	CSF2	0.3910
PRKACA	0.7084	UNG	0.0010	ERCC6	0.7749	CSF2RB	0.5235	SPRY2	0.9124
WNT2	0.2437	MBD4	0.2099	GTF2H5	0.0114	IL2RB	0.3012	STAT6	0.0748
SMO	0.0165	LIG1	0.1083	TAB1	0.7749	EP300	0.0615	IL5	0.8088
WNT5A	1.0000	POLD4	0.3012	MAPK11	0.0408	IL6	0.0615	IL13	0.5235
PRKX	0.6441	POLD3	0.1797	RIPK2	0.0129	CREBBP	0.0748	IL4	0.3442
GLI1	0.1405	POLD1	0.5526	UBB	0.1797	CCND2	0.0006	IL9	0.5235
ZIC2	0.4414	POLE	0.8776	MAP2K3	0.3442	IL5RA	0.8776	IFNG	0.2811
SHH	0.6760	APEX2	0.3671	MAPKAPK3	0.6441	SOCS1	0.9474	CSF3	0.7084
GSK3B	0.4414	POLE3	0.6760	TRAF6	0.1528	PTPN6	0.0263	JAK2	0.1405
FBXW11	0.2263	MUTYH	0.3671	RPS27A	0.0209	PRLR	0.5824	SOS1	0.2099
WNT11	0.4157	PARP1	0.0503	UBC	0.1943	IL7R	0.3012	CBLB	0.0263
GLI2	0.0235	MNAT1	0.8088	MAP2K6	0.0329	LIFR	0.1291	IL15RA	0.0030
PRKACG	0.9474	RPA3	0.4953	MAPK14	0.0453	OSMR	0.2619	STAT5B	0.2619
CSNK1D	0.2619	RFC1	1.0000	IRAK1	0.7414	PIAS4	0.3012	IL2RA	0.0903
LRP2	0.0022	ERCC4	0.3012	MAP3K7	0.0114	PIAS1	0.5824	STAT5A	1.0000
GAS1	0.3222	RPA1	0.3671	TAB2	0.0186	GHR	0.1591	SOCS5	0.0453
PTCH1	0.4157	XPC	0.8776	MAPKAPK2	0.7084	IL6ST	0.0012	STAT3	0.0557
WNT6	0.0557	RFC2	1.0000	IKBKG	0.2099	IL4R	0.0209	AKT2	0.6760
IHH	0.0990	ERCC8	0.4157	POLR2F	0.0557	AKT1	0.0823	SOCS2	0.3671
WNT8B	0.1797	RFC3	0.5824	POLR2E	0.9825	PIK3R1	0.3671	IFNB1	0.2099
BTRC	0.0114	CDK7	0.7749	POLR2L	0.0209	TPO	0.0030	IFNW1	0.4414
WNT4	0.8776	GTF2H2	0.7084	DICER1	0.1083	TYK2	0.9124	IFNA21	0.6760
PRKACB	0.0294	RAD23A	0.0114	POLR2B	0.2619	CTF1	0.0990	IFNA10	0.9825
WNT2B	0.3910	CCNH	0.5526	POLR2K	0.0040	EPOR	0.4953	IFNA16	0.8088
PARP2	0.0186	GTF2H1	1.0000	POLR2C	0.6129	EPO	0.9124	IFNA5	0.6441
APEX1	0.8088	DDB2	0.7084	POLR2J	0.9474	PIK3CG	0.2437	IFNA6	0.5975
POLE2	0.2619	DDB1	0.8088	TARBP2	0.0823	MYC	0.8776	IFNA2	0.6284
MPG	0.2437	CUL4A	0.0557	POLR2I	1.0000	IL2	0.0017	IFNA8	0.4414
NTHL1	0.2099	ERCC2	0.0679	POLR2G	0.4679	JAK3	0.6129	IFNA1	0.1183
POLD2	0.0263	ERCC1	0.4157	RAN	0.0078	SPRY1	0.2263	SPRED2	0.8431
PARP4	0.0101	RFC5	0.0366	POLR2H	0.0408	IL12RB1	0.3012	IL12B	0.5824
OGG1	0.5235	GTF2H3	0.2619	POLR2D	0.1405	PIK3R2	0.0030	PTPN11	0.0329
POLB	0.4952	ERCC3	0.7414	PRKRA	0.1658	IL15	0.1797	PIK3CB	0.0165
HMGB1	0.0235	RFC4	0.8431	IFNAR2	0.0366	CSF2RA	0.4157	STAM	0.4953
CNTFR	0.0679	STAT1	0.1797	PIK3R3	0.3222	CBL	0.0615	CNTF	0.8431
IL11RA	0.4953	STAT4	0.6129	JAK1	0.0557	IL2RG	0.9124	GH1	0.2437
CCND1	0.6441	IL13RA2	0.2811	IL12RB2	0.3222	IL9R	0.0408	IRF9	0.8776
BCL2L1	0.0235	IL13RA1	0.5235	PIAS3	0.0001	CSF3R	0.9825	IFNA4	0.0679
GH2	0.8431	PIM1	0.2099	IL6R	0.1083	IFNGR1	0.0146	IFNA14	0.9474
PRL	0.6441	CCND3	0.9124	IL10	0.0329	MPL	0.5823		
PIK3CA	0.0209	PIK3CD	0.0263	AKT3	0.9474	GRB2	0.0903		
SOCS3	0.5235	IL10RA	0.5235	IL11	0.6441	IL10RB	0.7749		

5. R code for statistical method AGCP

```
library(gtools)    ## permutations and combinations
################################################################################
###
################# Main Function    ##########################################
############################################################################
#### Input:
### x1: data from group 1
### x2: data from group 2
### cut.vec:    a set of specificed thresholds
#### Output: p-value of AGCP
#############################
AGCP <- function(x1, x2, cut.mat, ref.dis1, ref.dis2, ref.MAX.stat)
{
   m <- ncol(x1)
   ### calculate the mariginal p-values
   pval.vec <- rep(NA, m)
   for(i in 1:m)
   {
      pval.vec[i] <- wilcox.test(x1[,i], x2[,i])$p.value
   }
   ### the threhold matrix
   cut.mat <- cutpoint.fun(cut.vec)
   ### reference statistics
   temp.ref <- ref.ranktest(x1, x2, cut.mat, B)
   ref.dis1 <- temp.ref[[1]]
   ref.dis2 <- temp.ref[[2]]
   ref.MAX.stat <- temp.ref[[3]]
   #############################
   u <- nrow(cut.mat)
   stat.vec <- rep(NA, u)
   for(k in 1:u)
   {
      temp.cut <- cut.mat[k, ]
      loc1 <- which(pval.vec <= temp.cut[1])
      loc2 <- which(pval.vec > temp.cut[1] & pval.vec <= temp.cut[2])
      stat1 <- sum(-2*log(pval.vec[loc1]))
      stat2 <- sum(-2*log(pval.vec[loc2]))
      com1 <- mean(stat1 <= ref.dis1[,k])
      com2 <- mean(stat2 <= ref.dis2[,k])
      stat.vec[k] <- -2*log(com1)-2*log(com2)
   }
```

```
    MAX.stat <- max(stat.vec)
    pval_AGCP <- mean(ref.MAX.stat > MAX.stat)
    return(pval_AGCP)
}



################################################################################
###
############# Preliminary Functions
################################################################################
###
cutpoint.fun <- function(cut.vec)
{
    count.cut <- length(cut.vec)
    u <- count.cut*(count.cut-1)/2
    cut.mat <- matrix(NA, nrow=u, ncol=2)
    a <- 0
    for(i in 1:(count.cut-1))
    {
        for(j in (i+1):count.cut)
        {
            a <- a + 1
            cut.mat[a, ] <- c(cut.vec[i], cut.vec[j])
        }
    }

    cut.mat
}



ref.ranktest <- function(x1, x2, cut.mat, B)
{
    m <- ncol(x1)
    n1 <- nrow(x1)
    n2 <- nrow(x2)
    u <- nrow(cut.mat)
    ref.dis1 <- matrix(NA, nrow=B, ncol=u)
    ref.dis2 <- matrix(NA, nrow=B, ncol=u)

    temp.mat <- rbind(x1, x2)

    pval.mat <- matrix(NA, nrow=B, ncol=m)
```

```
    for(a in 1:B)
    {
        temploc <- sample(1:(n1+n2))
        loc1 <- temploc[1:n1]
        loc2 <- temploc[(n1+1):(n1+n2)]
        x1.boot <- temp.mat[loc1, ]
        x2.boot <- temp.mat[loc2, ]
        pval.mat[a, ] <- Wilcox.pval.vec(x1.boot, x2.boot)
        temp.pv <- sort(pval.mat[a,])
        for(k in 1:u)
        {
            c1 <- cut.mat[k, 1]
            c2 <- cut.mat[k, 2]
            loc1 <- which(temp.pv <= c1)
            loc2 <- which(c1 < temp.pv & temp.pv <= c2)
            stat1 <- sum(-2*log(temp.pv[loc1]))
            stat2 <- sum(-2*log(temp.pv[loc2]))
            ref.dis1[a, k] <- stat1
            ref.dis2[a, k] <- stat2
        }
    }

    ref.stat <- matrix(NA, nrow=B, ncol=u)

    MAX.stat <- rep(NA, B)
    for(a in 1:B)
    {
        for(k in 1:u)
        {
            com1 <- mean(ref.dis1[a,k]<=ref.dis1[, k])
            com2 <- mean(ref.dis2[a,k]<=ref.dis2[, k])
            ref.stat[a,k] <- -2*log(com1)-2*log(com2)
        }
        MAX.stat[a] <- max(ref.stat[a,])
    }

    list(ref.dis1, ref.dis2, MAX.stat)

}
```
